# White Blood Cell Count and the Risk for Coronary Artery Disease in Young Adults

**DOI:** 10.1371/journal.pone.0047183

**Published:** 2012-10-12

**Authors:** Gilad Twig, Arnon Afek, Ari Shamiss, Estela Derazne, Dorit Tzur, Barak Gordon, Amir Tirosh

**Affiliations:** 1 Department Medicine ‘B’, Chaim Sheba Medical Center, Tel-Hashomer, Israel; 2 The Dr. Pinchas Borenstein Talpiot Medical Leadership Program, Chaim Sheba Medical Center, Tel-Hashomer, Israel; 3 The Israel Defense Forces Medical Corps, Tel-Hashomer, Israel; 4 The Chaim Sheba Medical Center Management, Chaim Sheba Medical Center, Tel-Hashomer, Israel; 5 The Sackler School of Medicine, Tel-Aviv University, Tel-Aviv, Israel; 6 The Division of Endocrinology, Diabetes and Hypertension, Brigham and Women’s Hospital, Harvard School of Public Health, Boston, Massachusetts, United States of America; Innsbruck Medical University, Austria

## Abstract

**Background:**

The association between white blood cell (WBC) count and coronary artery disease (CAD) is unknown in young adults. Our objective was to assess the association between WBC count and its changes over time with CAD incidence in the Metabolic, Life-style and Nutrition Assessment in Young adults (MELANY) study, a cohort of Israeli army personnel.

**Methods and Findings:**

29,120 apparently healthy young men (mean age; 31.2±5.5 years) with a normal baseline WBC count (3,000–12,000 cells/mm^3^) were followed during a mean follow up of 7.5±3.8 years for incidence of CAD. Participants were screened every 3–5 years using a stress test, and CAD was confirmed by coronary angiography. In a multivariate model adjusted for age, body mass index (BMI), LDL- and HDL-cholesterol, blood pressure, family history of CAD, physical activity, diabetes, triglycerides and smoking status, WBC levels (divided to quintiles) above 6,900 cells/mm^3^ (quintile 4) were associated with a 2.17-fold increase (95%CI = 1.18–3.97) in the risk for CAD as compared with men in quintile 1 (WBC≤5,400 cells/mm^3^). When modeled as a continuous variable, a WBC increment of 1000 cells/mm^3^ was associated with a 17.4% increase in CAD risk (HR 1.174; 95%CI = 1.067–1.290, p = 0.001). A decrease in the WBC level (within the normal range) during the follow-up period was associated with increased physical activity and decreased triglyceride levels as well as with reduced incidence of CAD.

**Conclusions:**

WBC count is an independent risk factor for CAD in young adults at values well within the normal range. WBC count may assist in detecting subgroups of young men at either low or high risk for progression to CAD.

## Introduction

A growing body of evidence suggests that low grade inflammation contributes to the development of coronary artery disease [1]. In recent years, multiple markers of inflammation have been tested as potential risk factors for the development of CAD such as IL-6, E-selectin and CRP [2], [3]. Yet, none of these markers, including CRP, are routinely recommended for screening of apparently healthy subjects with intermediate risk for CAD [4].

Elevated white blood cell count (WBC) that is well within the normal range was associated with an increased risk for developing CAD [5], [6], or for the re-occurrence of myocardial ischemia [7], [8]. Yet, its application into CAD risk stratification in clinical practice is controversial for several reasons. First, the at-risk WBC cutoff values contain a wide range depending mostly on the characteristics of the population studied and the quality of adjustment for other risk factors [9]–[12]. Second, the potential interactions of WBC with the ‘classic’ CAD risk factors are largely unknown. Such interactions are important both for defining the relative contribution of WBC count to the development of CAD compared to known risk factors, as well as for identifying risk factors that can be amplified by low-grade inflammation. For example, an Australian cohort in middle aged men showed that WBC count greater than 6,900 cell/mm^3^ nearly doubled the CAD risk in patients with hypertriglyceridemia [13]. Finally, most prospective works used a single rather than repeated WBC count evaluations [10]–[12], [14]–[19]. This methodology raised practical questions on measurement reproducibility and the potential bias by intercurrent medical conditions which are not necessarily attributed to chronic inflammation and do not impose an excess cardiovascular risk.

In this study, we have used the comprehensive MELANY cohort of the Israeli Defense Forces Medical Corps to study whether WBC count can assist in predicting CAD among young and apparently healthy men. We report that WBC levels, well within the normal range, can reliably predict CAD in young men independently from the ‘classic’ cardiovascular risk factors.

## Research Design and Methods

### Study Population

The Metabolic, Life-Style, and Nutrition Assessment in Young Adults (MELANY) Cohort has been conducted at the Israel Defense Forces Staff Periodic Examination Center (SPEC) to which all career service personnel older than 25 years of age are referred every three to five year as described previously [20]–[24]. At each visit to the SPEC, participants completed a detailed questionnaire assessing demographic, nutritional, lifestyle, and medical factors. Thereafter, blood samples were drawn after a 14-hour fast and analyzed. Height, weight and blood pressure were measured, and a physician at the center performed a complete physical examination. Primary care for all Israel Defense Forces personnel between scheduled visits to the center is obtained at designated military clinics, and all medical information was recorded in the same central database, thereby allowing an ongoing, tight, and uniform follow-up.

Included in this study were 37,418 men who had documented WBC count within the normal range (3,000–12,000 cells/mm^3^) at their first visit to the SPEC between the years 1995 and 2010. Men with newly diagnosed CAD at baseline (n = 67) or men with a follow-up shorter than 2 years (n = 8,231) were excluded from analysis. We had data available for 3,968 women in our cohort, of which only 2 developed CAD, thus excluding the possibility for meaningful statistical analysis on the predictive value of WBC. Thus, 29,120 subjects remained with at least one measurement of WBC count with a follow up period exceeding 2 years. Of these, 18,479 subjects had at least two measurements of WBC (measured at least 2 years apart) allowing us to perform a sub analysis to evaluate the effect of repeated WBC measurements on CAD incidence.

The institutional review board of the Israel Defense Forces Medical Corps approved this study on the basis of strict maintenance of participants’ anonymity during database analyses.

### Follow-up and Outcome

Participants were followed prospectively from their first visit to the SPEC (mean age 31.2±5.5 years). Follow-up ended at the time of CAD diagnosis, death, retirement from military service or March 8^th^ 2011, whichever came first. Mean follow-up was 7.51±3.86 years.

The outcome definition for CAD in MELANY was angiography-proven stenosis of >50% in at least one coronary artery. Up to age 35, referral for a diagnostic procedure was based on specific complaints, and above 35 years of age all participants underwent a treadmill exercise test (Bruce protocol) in the presence of a board-certified cardiologist. Endpoints for the exercise test were ST-segment depression >2 mm in two contiguous leads, measured 80 milliseconds after the J-point, symptoms of angina, exhaustion, or achievement of the target heart rate. All participants with an abnormal stress test were referred for coronary angiography. When the stress test was borderline or when participants reported angina symptoms without diagnostic ECG changes, stress perfusion thallium-201 imaging was performed, followed by coronary angiography for participants with a pathological scan. Those presenting with symptoms of angina and/or MI between SPEC visits were referred for coronary angiography following consultation with a board-certified cardiologist.

### Statistical Analysis

The cohort population was divided into WBC quintiles and their baseline characteristics are presented in Table 1. The median of the quintiles were fit as continuous variables to estimate the trend of variables across quintiles in a linear regression model (adjusted R^2^ = 0.99, B = 909 cells/mm^3^ per quintile, p = 0.001). Cox proportional hazard models were used to estimate the Hazard Ratios (HR) and 95% confidence intervals (CI) for developing CAD. Following age-adjustment (model 1) additional known CAD risk factors were added in a stepwise manner (Table 2). In model 2, BMI was added to model 1 as a continuous variable. In model 3, smoking status (current-smoker, ex-smoker, never smoked) and physical activity (not active, <150 min/week, ≥150 min/week) were added to model 2 as categorical variables. In model 4, blood pressure (systolic <120 and diastolic <80; systolic ≥120 and/or diastolic ≥80; systolic ≥130 and/or diastolic ≥85; systolic ≥140 and/or diastolic ≥90 mmHg) was added as a categorical variable. In model 5, high-density lipoprotein cholesterol (HDL), low-density lipoprotein cholesterol (LDL), and serum triglyceride levels were added to model 4 as continuous variables. In model 6, occurrence of diabetes (yes/no) or family history of CAD (yes/no) were added as categorical variables. Log minus log plots for each variable were inspected to verify the assumption of proportionality of the hazards. All variables used in the model were tested for co-linearity using Pearson’s correlation. The maximal R recorded were 0.37 (triglyceride and BMI) and 0.28 (diastolic blood pressure and BMI). Omnibus tests of model coefficients were used to assess the relative contribution of the various variables to the model. In order to evaluate the power of the models to discriminate events from nonevents we calculated the area under the receiver operating characteristic (ROC) curve for each of the variables as well as in a multivariate model (C statistic). In a sub group analysis of 18,439 participants who had additional WBC measurements, the study population was divided into tertiles (low, intermediate and high) of WBC at baseline and once again following the second measurement. WBC tertiles of the first measurements were as follow; T1, WBC≤5,900 cells/mm^3^; T2, 5,900<WBC≤7,110; T3, WBC>7,110 cells/mm^3^, and the second measurement tertiles were: T1, WBC≤6,000 cells/mm^3^; T2, 6,000<WBC≤7,300; T3, WBC>7,300 cells/mm^3^. Therefore, for simplicity and clarity of presentation, we divided the tertiles of both measurements based on the WBC tertile range of the first measurement. Hazard ratios for CAD were estimated for the 9 cross-classified groups in a multivariate model adjusted for age, BMI, smoking status, physical activity, systolic and diastolic blood pressure, HDL, LDL, triglycerides, diabetes status and family history of CAD. The differences in metabolic parameters between first and second examinations were compared among the 4 subgroups of WBC (Table 3) with Dunnet-T3 test post-hoc multiple comparisons test. Reported values throughout the study are presented as mean± standard deviation (SD) unless mentioned otherwise. Analyses were performed with SPSS statistical software Vers.19.0.

**Table 1:** Baseline characteristics of population cohort.

Quintile of WBC count	Q1	Q2	Q3	Q4	Q5	Total or Average±SD	p
N	5,900	5,630	6,449	5,322	5,819	29,210	
WBC count (cells/mm^3^)	3,000–5,400	5,401–6,100	6,110–6,900	6,910–7,810	7,810–12,000	6,682±1,500	
Age±SD (years)	30.8±5.4	31.0±5.4	31.1±5.4	31.4±5.6	30.5±5.7	31.2±5.5	NS
BMI (kg/m^2^)	24.4±3.4	25.0±3.6	25.5±3.8	26.1±4.0	26.4±4.0	25.4±4.0	<0.001
BMI<25	63%	54%	48%	43%	36%	49%	
25≤BMI<30	31%	37%	40%	41%	43%	38%	
BMI≥30	6%	9%	12%	16%	21%	13%	
BPSystolic/BPDiastolic	115.5±11.9/	116.5±12.2/	117.2±12.4/	118±13.0/	119.4±13.4/	117.4±12.7/	
(mean±SD, mm Hg)	73.6±9.2	74.1±9.4	74.8±9.5	75.6±10.0	76.2±10.1	74.9±9.7	<0.001
Fasting glucose level	89.4±11.0	90.3±12.3	90.7±12.8	91.1±13.8	92.6±17.0	90.8±13.5	<0.001
HDL (mg/dL)	48.2±11.3	47.1±10.7	46.1±10.5	45.4±10.6	44.0±10.6	46.2±10.8	<0.001
LDL (mg/dL)	113.7±32.6	117.4±33.1	118.6±33.1	121.2±34.2	124.7±35.2	119.1±33.8	<0.001
Triglycerides (mg/dL)	100.8	111.5	123.3	135.2	153.3	124.7	
[25^th^; 75^th^]	[61;119]	[68;134]	[73;150]	[79;163]	[88;187]	[71;151]	<0.001
Physically active (%)	40.2 (8.0)	37.4 (8.0)	24.2 (8.2)	23.4 (7.1)	29.9 (6.0)	34.5 (7.5)	<0.001
Positive family history of CAD (%)	5.8	6.2	6.3	6.9	7.4	6.5	NS
Smoking							<0.001
Never	68%	65%	60%	55%	41%	58%	
Ex-smoker	13%	14%	15%	13%	11%	13%	
Current smoker	19%	21%	25%	32%	48%	29%	

Life style, physical and biochemical characteristic data is presented for 29,120 subjects. ‘Physically active’ refers to participants who have self-reported of being physically active; values in parentheses indicate the percentage of participants in each quintile reporting of at least 150 minutes of physical activity each week.

**Table 2:** Hazard ratio for developing coronary artery disease (CAD) across WBC quintiles.

Quintiles of WBC count	Q1	Q2	Q3	Q4	Q5	Total or mean±SD
N	5,900	5,630	6,449	5,322	5,819	29,210
WBC count (1000 cells/ml)	3,000–5,400	5,401–6,100	6,110–6,900	6,910–7,810	7,810–12,000	6,682±1,500
Total new cases of CAD	17	17	34	52	68	188
Mean follow-up (years)	7.75±3.86	7.68±3.89	7.51±3.85	7.46±3.89	7.15±3.78	7.51±3.86
Person years of follow-up	45,753	43,278	48,442	39,728	41,639	218,840
Incidence per 1000 person years	0.37	0.39	0.7	1.30	1.63	0.86
Model 1- Age adjusted						
Hazard ratio	1	1.040	1.839	3.254	3.831	
95%CI		0.531–2.038	1.027–3.292	1.881–5.626	2.250–6.523	
P		0.908	0.040	<0.0001	<0.0001	
Model 2- Age and BMI adjusted						
Hazard ratio	1	0.976	1.676	2.870	3.184	
95%CI		0.498–1.913	0.935–3.004	1.654–4.979	1.856–5.463	
P		0.943	0.083	<0.0001	0.0001	
Model 3- Age, BMI, smoking status, physical activity						
Hazard ratio	1	0.939	1.550	2.437	2.439	
95%CI		0.479–1.840	0.864–2.783	1.399–4.245	1.406–4.232	
P		0.854	0.142	0.002	0.002	
Model 4- Age, BMI, smoking status, physical activity, systolic and diastolic blood pressure						
Hazard ratio	1	0.935	1.527	2.323	2.321	
95%CI		0.477–1.833	0.851–2.741	1.332–4.502	1.336–4.033	
P		0.845	0.156	0.003	0.003	
Model 5- Age, BMI, smoking status, physical activity, systolic and diastolic blood pressure, HDL, LDL, triglycerides						
Hazard ratio	1	1.007	1.405	2.193	1.889	
95%CI		0.495–2.048	0.743–2.655	1.198–4.015	1.029–3.466	
P		0.985	0.296	0.011	0.040	
Model 6- Age, BMI, smoking status, physical activity, systolic and diastolic blood pressure, HDL, LDL, triglycerides, diabetes, family history of CAD						
Hazard ratio	1	0.983	1.446	2.173	1.837	
95%CI		0.483–2.000	0.765–2.732	1.188–3.977	1.002–3.370	
P		0.963	0.256	0.012	0.049	

Multivariate Cox-regression analysis was used to derive the hazard ratios under different adjustments to lifestyle, physical and biochemical parameters. Results summarize 29,120 subjects of whom 188 cases of CAD were diagnosed during 218,840 persons-years of follow-up.

**Table 3:** Metabolic changes associated with changes in repeated WBC measurement in 18,439 subjects.

	T1→T1				T1→T3				T3→T1				T3→T3			
	1^st^ test	2^nd^ test	Difference	p	1^st^ test	2^nd^ test	Difference	p	1^st^ test	2^nd^ test	Difference	p	1^st^ test	2^nd^ test	Difference	p
N	3986				701				412				3950			
WBC (cells/mm^3^)	5.01	5.02			5.4±0.4	8.0±0.8	2.5±0.9		8.0±0.9	5.4±0.5	2.6±1.0		8.5±1.0	8.6±1.1	0.1±1.3	
Age±SD (years)	32.1±5.4	37.2±4.9	5.1±1.7		30.2±4.9	35.8±4.5	5.6±1.8		32.9±5.6	38.0±5.1	5.1±1.7		32.5±5.5	37.7±5.1	5.2±1.8	
BMI (kg/m^2^)	24.6±3.4	25.6±3.6	1.0±1.8	<0.001	24.8±3.7	26.4±4.3	1.6±2.1	<0.001	25.6±4.1	26.3±4.1	0.7±2.0	<0.001	27.1±4.1	28.3±4.6	1.2±2.2	<0.001
Systolic BP (mm Hg)	115.2±11.9	117.6±11.4	2.4±14.3	<0.001	116.0±12.9	119.5±12.3	3.5±15.5	<0.001	117.8±14.4	118.5±11.5	0.6±14.1	NS	118.5±13.1	121.3±13.2	2.8±15.4	<0.001
Diastolic BP (mm Hg)	74.2±9.3	75.1±9.0	0.9±11.5	<0.001	74.0±9.7	75.9±9.2	1.9±11.8	<0.001	75.5±10.3	76.1±9.3	0.7±11.7	NS	76.4±10.2	77.7±9.6	1.3±12.4	<0.001
Fasting glucose (mg/dL)	90.3±11.4	90.1±13.8	0.2±13.1	NS	89.4±10.3	89.7±11.9	0.3±11.6	NS	91.8±13.8	91.7±17.7	0.05±15.4	NS	92.7±15.7	93.8±23.4	1.1±21.2	0.001
HDL (mg/dL)	48.0±11.3	45.6±10.7	−2.4±9.1	<0.001	46.7±11.0	44.6±10.1	−1.9±9.1	<0.001	47.4±11.7	45.7±10.4	−1.7±9.9	0.001	43.8±10.6	41.7±9.3	−2.1±8.8	<0.001
LDL (mg/dL)	116.3±32.7	120.6±31.8	4.3±25.4	<0.001	116.6±31.8	121.1±31.5	4.5±26.3	<0.001	121.6±33.2	123.3±31.1	1.7±28.0	NS	125.2±34.2	126.4±34.2	1.2±27.9	0.04
TG (mg/dL)	109.2±72.2	121.2±81.6	12.0±64.0	<0.001	106.1±66.7	137.9±88.1	31.7±78.1	<0.001	127.2±78.1	117.0±62.1	−10.1±63.9	0.002	154.7±106.5	171.1±112.8	16.4±97.4	<0.001
Current Smoking	18%	15%	−3%	<0.001	30%	27%	−3%	NS	28%	20%	−8%	<0.001	47%	41%	−6%	<0.001
Physically active	34% (5%)	32% (9%)	−2% (4%)	NS	40% (8%)	29% (9%)	−11% (1%)	<0.001	26% (9%)	40% (13%)	14% (4%)	<0.001	27% (4%)	26% (8%)	−1% (4%)	NS

WBC count range for each tertile (T) was determined by values at the first measurement: T1, WBC≤5,900 cells/mm^3^; T3, WBC>7,110 cells/mm^3^. ‘Physically active’ refers to participants who have self-reported of being physically active; values in parentheses indicate the percentage of participants in each quintile reporting of at least 150 minutes of physical activity each week.

## Results

### Characteristics of Study Participants

The 29,120 young men participated in this study were divided into quintiles based on their WBC count at enrollment. Baseline characteristics are presented in Table 1. Mean WBC count was 6,681±1,500 cells/mm^3^ (range 3,000–12,000 cells/mm^3^) with an average increment of 909 cells/mm^3^ between consecutive quintiles. WBC level was directly correlated with body-mass index (BMI), systolic and diastolic blood pressure, triglycerides level, LDL and rates of current smokers. Physical activity and HDL were inversely correlated with WBC levels (p<0.001).

### WBC Count is an Independent Risk Factor for Developing CAD

During 218,840 person-years of follow-up, 188 new cases of coronary artery disease (CAD) were diagnosed. The incidence of CAD increased linearly across quintiles of WBC, with 17 new cases diagnosed in the bottom quintile (Q1,WBC of 3,000–5,400 cells/mm^3^) and 68 new cases diagnosed in Q5 (WBC >7,810 cells/mm^3^). In model 1, adjusted for age, the hazard ratio (HR) for developing CAD was significant in the third quintile (Q3, 6,110<WBC<6,900 cells/mm^3^, HR = 1.83, 95%CI = 1.02–3.29, p = 0.04), reaching an HR of 3.83 in Q5 (95%CI = 2.25–6.52, p<0.001) as compared with Q1 (Table 2). While further adjustments for BMI (model 2) and smoking status (model 3) attenuated the risk for CAD, the risk attributed for increased WBC count remained significantly and independently elevated even in the multivariate model adjusted for age, BMI, smoking status, physical activity, blood pressure, HDL, LDL, triglycerides level, diabetes status and family history of CAD (model 6), starting at levels >6,910 cells/mm^3^ (model 6, HR observed in Q4 vs. Q1, 2.17; 95%CI = 1.18–3.97). When WBC count was modeled as a continuous variable, an increment of 1000 cells/mm^3^ in WBC count was associated with a 17.4% increase in the risk for developing CAD independently of age, BMI, smoking status, physical activity, blood pressure, HDL, LDL, triglycerides level, diabetes status and family history of CAD (HR 1.174; 95%CI = 1.067–1.290, p = 0.001).

### Risk Prediction of CAD in Young Adults using WBC Count

**Figure 1:**
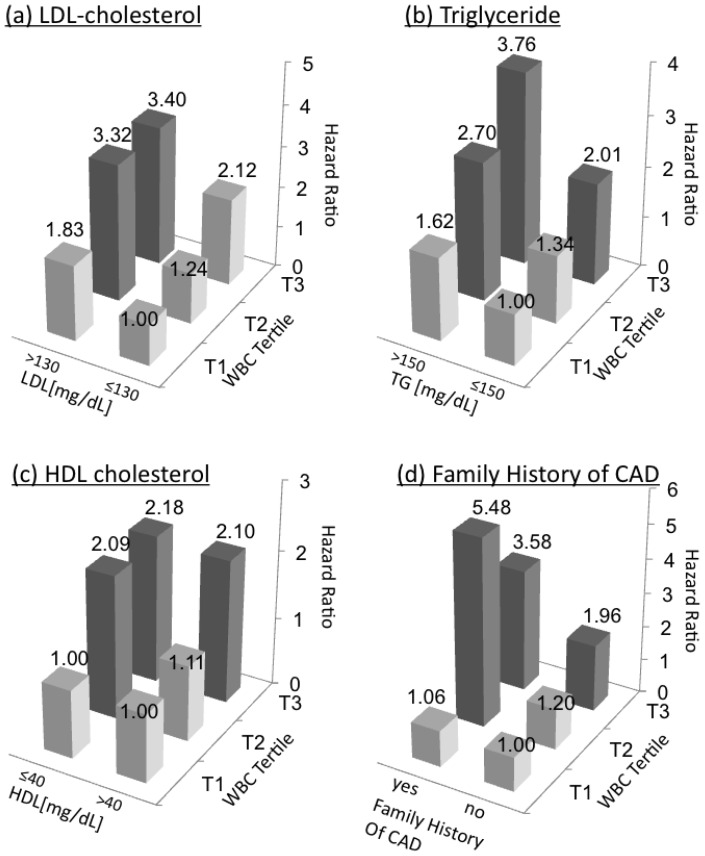
The joint effect of WBC tertiles and established risk factors for CAD. WBC tertiles were divided as followed: T1, WBC≤5,900 cells/mm^3^; T2, 5,900<WBC≤7,110; T3, WBC>7,110 cells/mm^3^. The reference group in all tests was T1. Multivariate models were adjusted to age, BMI, smoking status, physical activity, systolic and diastolic blood pressure, LDL, HDL, triglyceride level, diabetes status and family history of CAD. P value of interactions for WBC with LDL, triglyceride level or family history of CAD were 0.510, 0.951 and 0.123, respectively. Dark bars indicate p<0.05.

In order to better assess the interrelation between altered blood lipid profile and WBC, we next studied the joint effect of LDL and WBC levels in predicting the risk for developing CAD (Fig. 1a). When LDL levels were cross-classified with WBC tertiles in a multivariate analysis controlled for all of the covariates used in model 6, men with LDL level greater than 130 mg/dL who were also at the upper WBC tertile (WBC>7,110 cells/mm^3^) had a 3.4-fold increase in the risk for CAD (95%CI = 1.57–7.32) as compared to the reference group. However, CAD incidence among participants with an elevated LDL level (>130 mg/dL) but with low WBC count (Tertile 1; WBC≤5,900 cells/mm^3^), was not significantly greater than the reference group (HR 1.83; 95%CI = 0.74–4.49; p = 0.18).

We next studied the joint effect of WBC with HDL, triglyceride level and family history of CAD in multivariate models (Fig. 1b–d). Similar to the observations with LDL cholesterol, lower WBC count (WBC≤5,900 cells/mm^3^) in young adults was again associated with CAD incidence that was not significantly increased, even in the presence of high triglyceride level (TG≥150 mg/dL, HR = 1.62, 95%CI;0.72–3.61, p = 0.24), low HDL (HDL<40 mg/dL, HR = 1.00, 95%CI;0.44–2.25, p = 0.99), or positive family history for CAD (HR = 1.06, 95%CI;0.25–4.52, p = 0.93) (Fig. 1b–d). Moreover, subjects with WBC at the upper tertile, even in the absence of high triglyceride levels, low HDL or positive family history of CAD, had an approximately 2-fold increase in the risk for CAD (p<0.03 after adjustment for the covariates listed in model 6) as compared to the lower WBC tertile (Fig. 1b–d).

### Residual Contribution of WBC to CAD Prediction

In order to assess the differential contribution of the various risk factors to the prediction of CAD, we next calculated the receiver operating characteristic (ROC) curves for both WBC count as well as for other, well-validated CAD risk factors. Surprisingly, the area under the curve (AUC) of the WBC count ROC curve was not statistically different than that of HDL (0.663 [95%CI = 0.625–0.702] vs. 0.627 [95%CI = 0.585–0.668], respectively), BMI (0.668 [95%CI = 0.632–0.704]) or systolic blood pressure (0.603 [95%CI = 0.560–0.646]). In a C-statistics analysis, addition of the covariates in model 6 to the age adjusted model (model 1) increased the AUC from 0.747 [95%CI = 0.720–0.774] to 0.864 [95%CI = 0.840–0.892]. In the full multivariate model, addition of WBC did not significantly increase the AUC (0.868 [95%CI = 0.843–0.894]). Of interest, none of the co-variates when added to the multivariate model could significantly improve the AUC of the ROC curve (data not shown).

We next assessed the change in -2 log likelihood in a Cox regression model, which unlike the C-statistics analysis, controls for variations in the duration of follow-up, with a forward stepwise addition of the variables (Omnibus test of model coefficients). The major contributor for the model was age followed by triglyceride levels, LDL, smoking status, presence of diabetes, family history of CAD, systolic blood pressure and WBC count. Of interest, BMI, HDL-cholesterol and physical activity contributed less to the prediction model as compared to WBC count.

### The Value of Repeated WBC Counts as a Risk Factor for CAD

Of the 29,120 subjects described in Table 1, 18,439 subjects had at least two WBC counts available, measured at least 2 years apart (mean 5.7±1.8 years) with a subsequent follow-up of 9.4±3.3 years. Of these, 81 participants were diagnosed with CAD during the follow-up period. As compared with the baseline WBC measurement, most subjects remained in the same relative tertile at the second test (57%), and in over 90% of cases the difference between the second to the first WBC count was less than 2,000 cell/mm^3^ (the Pearson’s correlation between first and second tests was 0.640, p<0.001). As assessed using the full multivariate model, subjects with a baseline WBC count at the upper two tertiles (T2 or T3) who were found to have a second WBC at the lower tertile (T1) had a complete reversal of their risk for CAD (HR_T2→T1_ = 0.793 [95%CI; 0.15–3.94, p = 0.77], HR_T3→T1_ = 0.857 [95%CI;0.09–7.44, p = 0.88]) as compared with participants who had a low WBC count at both time points (_T1→T1_). Subjects who were at the highest WBC tertile throughout the entire follow-up period have reached over 3-fold increase in the risk for CAD (HR 3.31; 95%CI;1.35–8.10, p = 0.009). The most recent WBC count (taken at the second time point, approximately 5 years following baseline measurements) was found to be an independent risk factor for incident CAD when added to the full multivariate model (model 6), p = 0.041, while the increased risk attributed to the baseline WBC measurement was completely attenuated (p = 0.831).

We next characterized the participants whom WBC counts at the second time point has changed between the two extreme tertiles (_T1→T3_ or _T3→T1_, Table 3). Groups were compared to subjects that remained in the lower or upper tertiles at the two time points (_T1→T1_ and _T3→T3_, respectively). Consistent with the cohort effect, all groups had a significant increase in BMI from time 1 (baseline) to time 2 (second measurement of WBC) as well as a reduction in HDL cholesterol levels. The T3→T1 group had the lowest weight gain (ΔBMI = 0.7±2.0 kg/m^2^) and the lowest increase in systolic blood pressure (0.6±14.1 mmHg) during follow-up, (p<0.03 for both, as compared with the other groups). In addition, the group of participants with an observed decrease in WBC count from T3 to T1 was the only group that exhibit a significant decrease in mean triglyceride level from 127.2±78.1 to 117.0±62.1 mg/dL (p = 0.002), while differences in LDL cholesterol or diastolic blood pressure were similar between subgroups. Participants demonstrating an increase in WBC count from the lowest to the highest tertile (_T1→T3_) had the highest increase in both weight (ΔBMI = 1.6±2.1 kg/m^2^), systolic and diastolic blood pressure (Δ = 3.5±15.5 and 1.9±11.8 mmHg, respectively), LDL-cholesterol (Δ = 4.5±26.3 mg/dL) and triglycerides (Δ = 31.7±78.1 mg/dL), (p<0.001 for all).

## Discussion

This large-scale follow-up study demonstrated that a single measurement of WBC in healthy young men may predict CAD incidence independently from other risk factors for CAD such as elevated lipids, and a positive family history. WBC level above 6,900 cells/mm^3^ was associated with a ∼2-fold increase in the risk for CAD with a significant 17.4% increase in CAD incidence observed for every increment of 1,000 WBC/mm^3^.

Previous studies assessing the relationship between WBC and CAD incidence are available mainly for middle aged participants and in the elderly population, with conflicting results. The HRs reported are usually between 1.1 to 2.0 [10]–[12], [15], [16], [18] with a reported ‘threshold’ for excess risk ranging from 6,600 to 9,200 leukocytes/mm^3^. Two major contributors to this variability could be recognized: an inconsistent adjustment to known risk factors including BMI, diabetes and altered lipid profile [4], and variable definitions of the cardiovascular outcomes. The definition of coronary disease in most studies was primarily self-reported or relied on clinical symptoms using different diagnostic criteria [5], [25]. Furthermore, it is likely that contribution of low-grade inflammation to CAD development is different along the stages of plaque formation [18]. Therefore, studying different ages at various stages in the natural history of atherosclerosis may lead to different results. It is possible that increased inflammation may be important at early stages in the natural history of coronary disease, while later in the course of disease progression, other risk factors may become more predominant. In this study, we used a pro-active screening approach in a young and apparently healthy population, with a unifying and accurate definition of CAD as assessed by coronary angiography. This allowed us to assess the role of WBC count relatively early in the course of coronary atherosclerosis, before symptoms develop as is usually the case at early ages. In addition, available to us was a comprehensive data set of other CAD risk factors, thus allowing us to conduct detailed adjustments to other known risk factors of CAD.

To the best of our knowledge, this is the first study using consecutive WBC counts rather than a single baseline measurement. In the context of CAD prediction, using multiple WBC measurements over time may be of benefit. First, the overall good reproducibility of the results over a period of several years underlies the validity of WBC count as a marker for chronic inflammatory burden assessment in healthy individuals. Furthermore, changes in WBC over time were accompanied by changes in both lifestyle parameters, anthropometric measurements and other metabolic markers, reflecting a ‘true’ change in CAD risk profile.

Several limitations of this study warrant consideration. First, the MELANY cohort may be considered representative of a unique group of healthy young men. However, the characteristics of the population are strikingly similar to those of cohorts in published studies of young men from various industrialized countries [26]–[29]. In addition, the relatively homogeneous environment to which participants in our study were exposed might reduce the effect of unknown confounders. Second, although they did not compromise the outcome definition, measurements of WBC differentials were not obtained in this study, limiting our ability to assess specifically the role of neutrophiles and monocytes as potentially more specific predictors for the development of CAD [25]. In addition, the relatively low rate of CAD incidence in this age group may compromise the statistical power to conclude for specific interactions between WBC and additional risk factors, as well as with repeated measurements of WBC. Furthermore, as mentioned above, the relevance of the proposed association between WBC and CAD should also be assessed for clinically overt cardiovascular outcomes. The strengths of the MELANY study include the detailed, uniform, and systematic follow-up and outcome definition; the use of measured (rather than reported) values for BMI calculation; the availability of reliable determinations of blood tests and the direct measurements of lipids.

In summary, high-normal WBC count, measured in young, healthy men in an outpatient screening setting, is an independent and reliable risk factor for CAD. The joint effect of WBC count, a readily available measurement, with other known risk factors for CAD may help to better identify young men at either high or low cardiovascular risk. Additional studies in other, more heterogeneous populations as well as in clinically overt cardiovascular disease are required in order to conclude about the potential use of WBC as a risk predictor for CAD.
